# Direct RNA sequencing identified solute carrier family 2 member 1 to improve neurological outcome prediction after cardiac arrest

**DOI:** 10.1186/s40635-025-00851-8

**Published:** 2026-01-07

**Authors:** Victoria Stopa, Miron Sopic, Lu Zhang, Andrew Lumley, Pascal Stammet, Claudia Schrag, Ondrej Smid, Christian Hassager, Jesper Kjaergaard, Tommaso Pellis, Janneke Horn, Michael Kuiper, Jan Hovdenes, Christian Rylander, Matt P. Wise, Niklas Nielsen, Yvan Devaux

**Affiliations:** 1 Cardiovascular Research Unit, Department of Precision Health, Luxembourg Institute of Health, Strassen, Luxembourg; 2 Department of Medical Biochemistry, Faculty of Pharmacy, University of Belgrade, Belgrade, Serbia; 3 Bioinformatics and AI Unit, Department of Medical Informatics, Luxembourg Institute of Health, Strassen, Luxembourg; 4 Department of Anesthesia and Intensive Care Medicine, Centre Hospitalier de Luxembourg, Strassen, Luxembourg; 5 Department of Life Sciences and Medicine, Faculty of Science, Technology and Medicine, University of Luxembourg, Esch-sur-Alzette, Luxembourg; 6 Medizinische Intensivstation, Kantonsspital St. Gallen, St. Gallen, Switzerland; 7 2nd Department of Medicine, Department of Cardiovascular Medicine, First Faculty of Medicine, Charles University in Prague and General University Hospital in Prague, U Nemocnice 2, 128 00 Prague 2, Czech Republic; 8 Department of Cardiology, Rigshospitalet and Dept of Clinical, Medicine, University of Copenhagen, Copenhagen, Denmark; 9 Departement of Emergency and Intensive Care, Azienda Sanitaria Friuli Occidentale, Via Montereale, 33170 Pordenone, Italy; 10 Dept of Intensive Care, Amsterdam UMC, Amsterdam, The Netherlands; 11 Amsterdam Neuroscience, University of Amsterdam, Amsterdam, The Netherlands; 12 Department of Intensive Care, Frisius Medical Center, Leeuwarden, Netherlands; 13 Department of Anesthesia and Intensive Care, Oslo University Hospital, Rikshospitalet, Oslo Norway; 14 Anaesthesiology and Intensive Care Medicine, Department of Surgical Sciences, Uppsala University and Uppsala University Hospital, 715 85 Uppsala, Sweden; 15 Adult Critical Care, University Hospital of Wales, Cardiff, UK; 16 Department of Clinical Sciences Lund, Anesthesia and Intensive Care, Helsingborg Hospital, Lund University, Svart‑brödragränden 3, 251 87 Helsingborg, Sweden

**Keywords:** Cardiac arrest, Neurological outcome, Prognostic biomarker

## Abstract

**Background:**

Cardiac arrest (CA) is a major cause of mortality and morbidity. Accurate prediction of neurological outcome and survival remains challenging. In this context, our study aimed to explore novel molecular biomarkers that could provide additional insights into the pathophysiology of brain injury after CA and potentially distinguish patients with no brain injury (CPC 1) from those with any degree of neurological damage from moderate injury up to death (CPC 2–5), and complement existing prognostic tools.

**Methods:**

Whole blood samples collected 48 h after return of spontaneous circulation were analyzed by RNA sequencing in a subgroup of 50 CA patients from the monocenter North Pole cohort, and by quantitative PCR in 233 patients from the same cohort as well as in 511 patients from the multicenter TTM trial. The association of gene expression changes with 6-month neurological outcome (assessed by the Cerebral Performance Category (CPC) score) and survival was studied.

**Results:**

In a discovery phase with a subset of 50 patients from the North Pole cohort (25 CPC 1 and 25 CPC 5), direct RNA sequencing identified the solute carrier family 2 member 1 (SLC2A1), a gene encoding a major glucose transporter at the blood–brain barrier (GLUT1), as significantly upregulated in CPC 5 patients (dead with severe neurological impairment) compared to survivors without neurological sequelae (CPC 1). This upregulation was confirmed by quantitative PCR and extended to the entire North Pole cohort (*p* < 0.001). SLC2A1 was an independent predictor of neurological sequelae or death in this cohort. In the TTM trial, SLC2A1 was also upregulated in patients with neurological sequelae or death (*p* < 0.001) and was an independent predictor of neurological sequelae or death, providing an incremental predictive value to a baseline clinical model (odds ratio = 2.06, 95% confidence interval 1.31–3.4, *p* = 2.82E-03, and likelihood ratio test *p* < 0.001).

**Conclusion:**

Blood level of SLC2A1 is a tentative blood biomarker that may aid in neurological outcome prediction after CA and also provide new insights into post-CA injury mechanisms.

**Supplementary Information:**

The online version contains supplementary material available at 10.1186/s40635-025-00851-8.

## Introduction

Cardiac arrest (CA), defined as the abrupt cessation of cardiac function, remains a global public health issue, responsible for around 20% of adult deaths in the United States and Western Europe [1]. Despite progress in advanced life support, post-resuscitation care, and public education initiatives, overall survival rates remain very low [2]. In Europe overall survival rates after CA average around 8% varying from 0 to 18% depending on factors such as the location, comorbidities and the healthcare system organization [3]. Among those who do survive, the majority achieve a favorable neurological outcome, with approximately 70–80% showing good recovery Cerebral Performance Category (CPC) scale 1–2 at 6 months [4]. Accurately predicting neurological outcome after CA remains a major clinical challenge. Prognostication relies on a multimodal approach that includes clinical examination, neurophysiology, neuroimaging, and a limited number of biomarkers, with neuron-specific enolase (NSE) being the only one currently recommended for clinical use [5, 6].

However, currently available biomarkers still have limitations in precision and consistency, which can make clinical interpretation challenging. Moreover, they do not fully capture the complex and evolving pathophysiology of ischemia–reperfusion injury, which underlies much of the neurological damage in these patients. Hypoxic–ischemic brain injury involves a cascade of molecular and cellular processes such as oxidative stress, inflammation, excitotoxicity, apoptosis, and disruption of the blood–brain barrier [4, 7, 8]. Given the complexity of this hypoxic–ischemic brain injury following CA, identifying biomarkers that reflect the underlying biological processes is essential. Recent advances in molecular profiling technologies, particularly in the field of transcriptomics, provide new opportunities to identify such biomarkers by uncovering gene expression changes associated with brain injury, inflammation, and recovery [9, 10]. By analyzing the transcriptional landscape of affected tissues or circulating RNAs, transcriptomic approaches can shed light on the molecular pathways involved in brain injury progression and potentially reveal novel prognostic biomarkers [11].

To identify novel transcriptomic biomarkers of neurological outcome and survival after CA, we analyzed whole blood samples from two independent cohorts of CA patients. In particular, we investigated the solute carrier family 2 member 1 gene (SLC2A1), which encodes the glucose transporter 1 (GLUT1), a key mediator of glucose transport across the blood–brain barrier [12].

## Material and methods

### Study populations

The North Pole cohort is a prospective observational study conducted in Luxembourg [13]. 233 adult patients who suffered a CA between 2008 and 2020 and for whom blood samples were available were enrolled in this study. Patients were admitted to the intensive care unit following either out-of-hospital or in-hospital CA. The study received approval from the National Ethics Committee of Luxembourg (CNER N°200,803/05), and written informed consent was obtained from patients or their legal representatives, in compliance with the Declaration of Helsinki and national regulations.

The TTM trial enrolled 950 CA patients from 10 countries between 2010 and 2013 [14]. Blood samples from 511 patients were available for the present study. The trial aimed to evaluate the impact of targeted temperature management at 33 °C or 36 °C on survival rates and neurological outcome after CA. Ethical approval procedures followed national regulations, and consent was either waived or obtained from patients or their families, following the Declaration of Helsinki. The study is registered at ClinicalTrials.gov (NCT01020916). Lactate and neuron-specific enolase (NSE) levels were measured 48 h after return of spontaneous circulation, as described previously [13, 14].

### End points

The primary end-point of this study is neurological outcome, as evaluated 6 months post-CA using the CPC score. A CPC score of 1 corresponds to a good neurological recovery without sequelae or with mild neurological deficit, whereas scores from 2 to 5 indicate varying degrees of neurological sequelae, from moderate up to CPC 5, corresponding to death. The aim of the study was indeed to distinguish patients with no or mild brain injury (CPC 1) from those with any degree of neurological damage ranging from moderate up to death (CPC 2–5), thereby capturing the full spectrum of post-CA brain injury. Although CPC 2 (moderate cognitive impairment) is generally considered a good neurological outcome, it was included within the unfavorable group to emphasize differences between patients with preserved brain function and those with residual or severe injury. It is acknowledged that CPC 5 (death) can sometimes result from non-neurological causes. The secondary end-point of the study was survival at 6 months.

### Blood sample collection and processing

Peripheral whole blood samples in both patient populations were drawn 48 h after the return of spontaneous circulation (ROSC) in RNA-stabilizing PAXgene blood RNA tubes. Total RNA isolation was carried out using the PAXgene blood RNA kit, according to the manufacturer’s instructions. RNA quantity was measured using Nanodrop spectrophotometer and RNA integrity was assessed using capillary electrophoresis.

### RNA sequencing (RNAseq)

Among the 233 patients of the North Pole cohort, a subset of 50 patients was used in a discovery phase with RNAseq, composed of 25 CPC 1 patients and 25 CPC 5 patients (Table 1), selected to represent the two extreme phenotypes of clinical outcome. RNA libraries were prepared using the Oxford Nanopore Technologies (ONT) Direct RNA Sequencing following standard kit protocol. Libraries were loaded onto an RNA chemistry flow cell and sequenced on a MinION device.

**Table 1:**
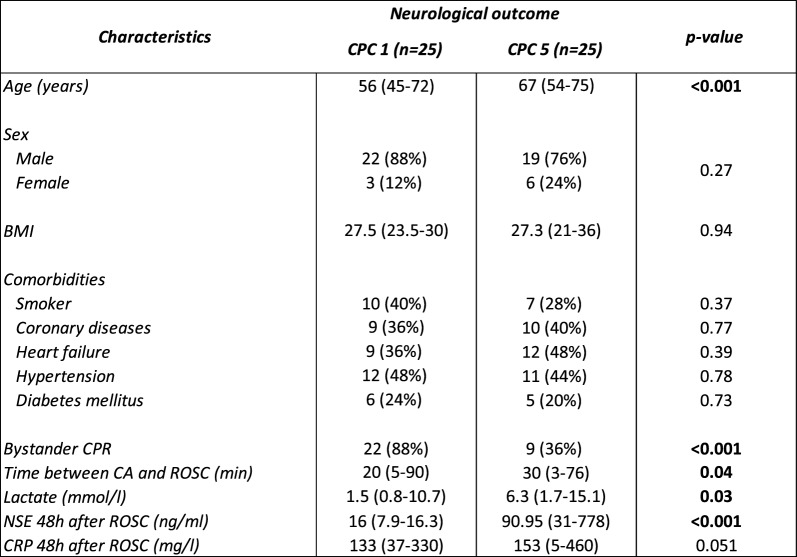
Characteristics of the 50 North Pole patients used for the discovery phase with RNAseq

Patients with poor neurological outcome (CPC 5) were older, less likely to receive bystander CPR, had longer time to ROSC, higher lactate, and markedly elevated NSE levels compared with CPC 1 survivors. Categorical variables are presented as number (proportion) and continuous variables are indicated as median (range). Body mass index (BMI), cardiopulmonary resuscitation (CPR), return of spontaneous circulation (ROSC), cardiac arrest (CA), neuron-specific enolase (NSE), C-reactive protein (CRP)

### Bioinformatic analysis of RNAseq data

Raw signal data were basecalled using Dorado (v0.8) to convert electrical signals into nucleotide sequences. High-quality reads (*Q*-score ≥ 7) were retained for downstream analysis. Basecalled reads were aligned to the reference genome (Gencode v44, GRCh38) using Minimap2 (v2.28) with options “-ax splice -u f -k 14” recommended for Nanopore direct RNA-seq. Gene counts were summarized from the transcript-level quantification using Bambu (v3.4) R package, and differential gene expression analysis was conducted using the DESeq2 (v1.42) R package. Gene representative analysis was performed using The Database for Annotation, Visualization, and Integrated Discovery (DAVID) web tool.

### Reverse transcription and quantitative PCR (qPCR)

For the North Pole cohort, 500 ng of total RNA per sample was used with High-Capacity Reverse Transcriptase and fothe TTM trial cohort, 300 ng of RNA per sample was processed using the SuperScript™ II Reverse Transcriptase. In both cases, random primers and dNTPs were added to reactions as described in the supplier’s protocol. The resulting cDNA was diluted 10-fold prior to quantitative PCR.

Quantitative PCR primers were designed using Primer3 (version 4.1.0) (https://primer3.ut.ee) and were as follows: SLC2A1-Forward: GTGGCCTTCTTTGAAGTGGG and SLC2A1-Reverse: AAGACGTAGGGACCACACAG, 18S-Forward: CGGCGACGACCCATTCGAAC and 18S-Reverse: GAATCGAACCCTGATTCCCCGTC, SF3A1-Forward: GATTGGCCCCAGCAAGCC and SF3A1-Reverse: TGCGGAGACAACTGTAGTACG. Each 20 µL qPCR reaction contained 10 µL of 2 × SYBR Green Master Mix, 0.6 µL of each primer (10 µM), 4 µL of cDNA template, and 4.8 µL of nuclease-free water. All reactions were performed in duplicate to ensure technical reproducibility. An internal run calibrator consisting of a pooled cDNA sample derived from PAXgene blood RNA tubes from CA patients was included in each qPCR plate. This calibrator enabled normalization of Ct values across plates and improved comparability of gene expression data. Negative controls (no-template controls, no-enzyme controls) were included in each run to monitor potential contamination or non-specific amplification. Amplification specificity was confirmed by melt curve analysis and by the absence of signal in negative controls. qPCR reactions were run on a CFX96 Real-Time PCR Detection System.

Gene expression was normalized using 18S ribosomal RNA (18S rRNA) in the North Pole cohort and splicing factor 3A subunit 1 (SF3A1) in the TTM1 trial cohort. These genes were used as reference genes, as they demonstrated the highest stability and lowest variability across samples and clinical conditions within their respective cohorts. Relative expression was calculated using the ΔΔCt method with Bio-Rad CFX Maestro 1.1 software (version 4.1.2433.1219), followed by log2 transformation and scaling for downstream analysis.

### Statistical analysis

Statistical analysis was performed using SigmaPlot 16.0 and R Studio. Data distribution was assessed using the Shapiro–Wilk test. For normally distributed variables, comparisons between groups were performed using the Student’s *t*-test, while non-normally distributed variables were compared using the Mann–Whitney *U* test. Categorical variables were analyzed using the Chi-squared test (or Fisher’s exact test when appropriate). SLC2A1 expression was used as a continuous variable in both univariate and multivariable logistic regression analyses to assess its association with 6-month neurological outcome, and in Cox proportional hazards models to evaluate its association with 6-month survival (supplementary figures). In both cohorts, neuron-specific enolase (NSE) levels at 48 h were included as a covariate in all multivariable analyses to adjust for their known prognostic value and minimize confounding. For Kaplan–Meier survival curves, SLC2A1 was dichotomized according to the Youden Index cutoff to visualize survival differences between groups (supplementary figures). The incremental predictive value of SLC2A1 to a baseline model was assessed using the Akaike information Criterion (AIC), the likelihood ratio test (lrt), the area under the receiver operating characteristic curve (AUC), the net reclassification improvement (NRI), and the integrated discrimination improvement (IDI). Different R packages were used in the analysis. Missing data were imputed using the MissForest package. The packages Hmisc and ROCR were used to compute the incremental predictive characteristics (AIC, LRT, AUC, NRI, and IDI).

## Results

### Patient demographics and clinical data

Demographic and clinical data, including age, sex, body mass index (BMI), comorbidities, bystander cardiopulmonary resuscitation (CPR), time from CA to return of spontaneous circulation (time to ROSC), lactate, neuron-specific enolase (NSE), and C-reactive proteins (CRP) levels were assessed across the two study populations and are summarized in Tables 1, 2, 3.

**Table 2:**
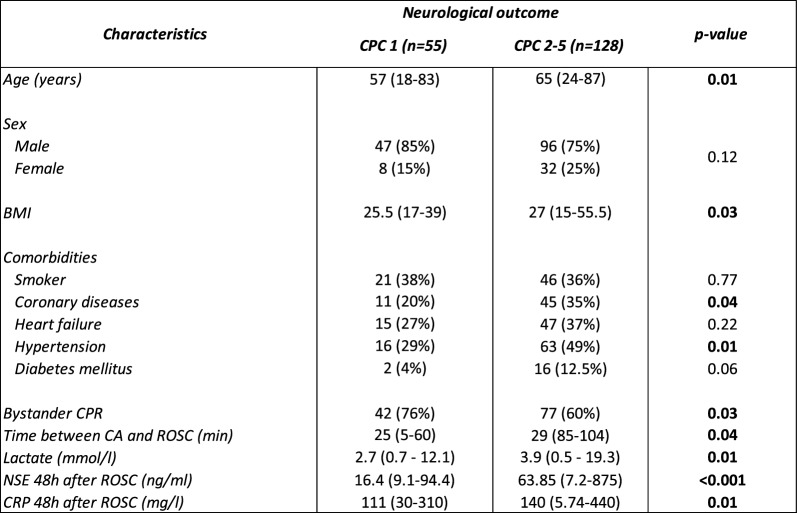
Characteristics of the 183 North Pole patients

Compared with CPC 1 patients, patients with CPC 2–5 outcomes were older, had higher BMI, were less likely to receive bystander CPR, had longer time to ROSC, higher lactate and CRP levels, higher NSE concentrations, and more frequently presented with comorbid coronary disease or hypertension. Categorical variables are presented as number (proportion) and continuous variables are indicated as median (range). Body mass index (BMI), cardiopulmonary resuscitation (CPR), return of spontaneous circulation (ROSC), cardiac arrest (CA), neuron-specific enolase (NSE), C-reactive protein (CRP)

**Table 3:**
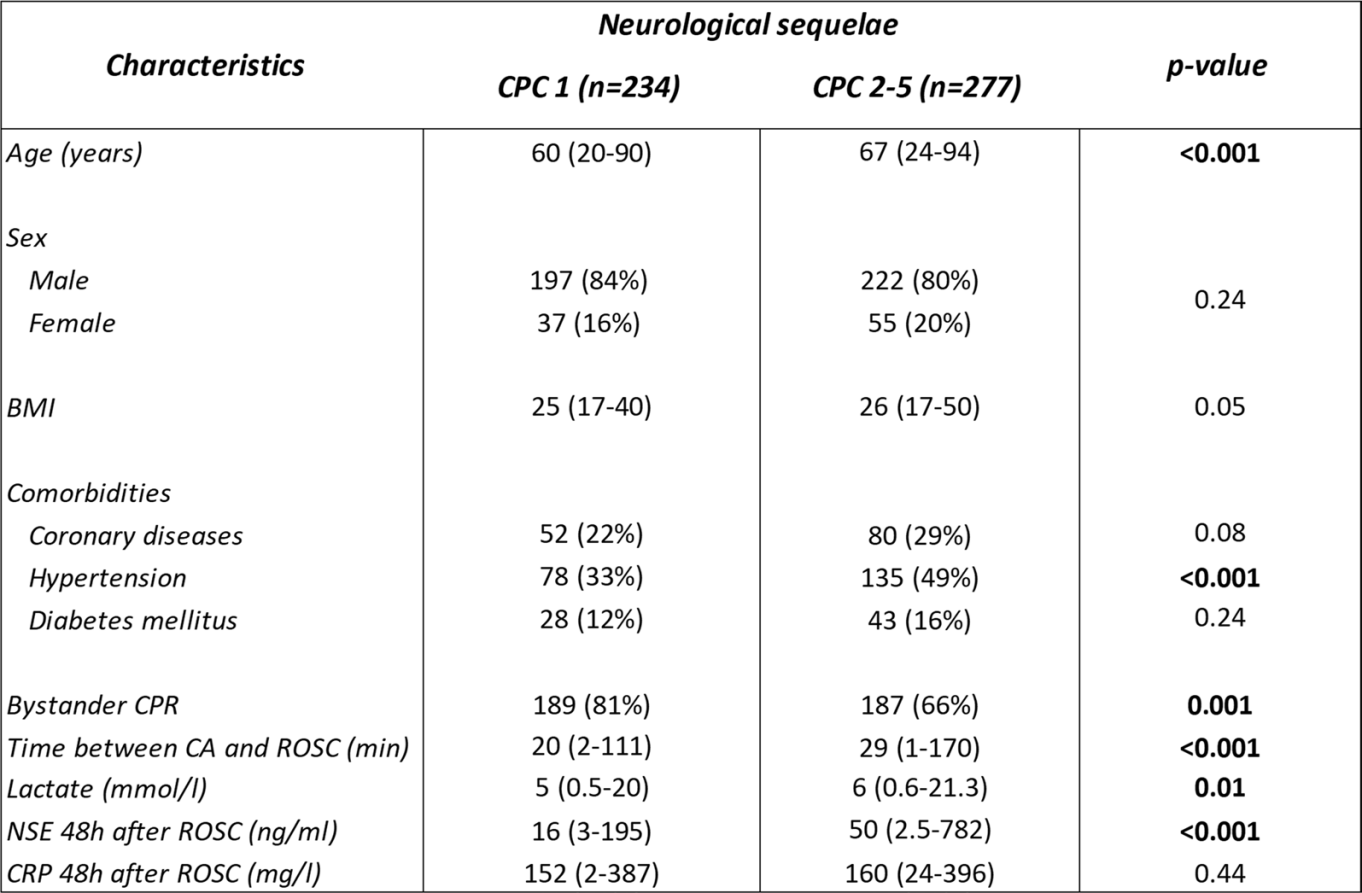
Characteristics of the 511 patients of the TTM trial

In the TTM trial, compared with CPC 1 patients, patients with neurological sequelae (CPC 2–5) were older, more frequently hypertensive, less likely to receive bystander CPR, had longer time to ROSC, higher lactate levels, and markedly elevated NSE concentrations. Categorical variables are presented as number (proportion) and continuous variables are indicated as median (range). Body mass index (BMI), cardiopulmonary resuscitation (CPR), return of spontaneous circulation (ROSC), cardiac arrest (CA), neuron-specific enolase (NSE), C-reactive protein (CRP)

Table 1 reports clinical data from the discovery cohort of 50 North Pole patients analyzed by RNAseq. This subgroup included 25 patients with good neurological outcome (CPC 1) and 25 patients who died (CPC 5), selected to represent the two extreme phenotypes of clinical outcome. Patients who died (CPC 5) were older as compared to patients with good neurological outcome. The two outcome groups, showed similar sex distribution and BMI. Similarly, the prevalence of common comorbidities (smoker status, coronary diseases, heart failure, hypertension, and diabetes mellitus) was comparable between groups. Patients of the CPC 1 group had more often received bystander CPR (88%) compared to patients of the CPC 5 group. Time to ROSC was shorter in the CPC 1 group compared to the CPC 5 group. Lactate and NSE levels measured 48 h after ROSC were lower in the CPC 1 group. CRP levels were of borderline significance.

Table 2 summarizes the clinical and demographic data of the 183 remaining patients of the North Pole cohort after removal of the 50 patients used in the discovery phase. This cohort was used in qPCR experiments and included 55 survivors without neurological sequelae (CPC 1) and 128 patients with neurological sequelae or death at 6 months (CPC 2–5). CPC 2–5 patients were older than CPC 1 patients and had a higher BMI. While sex distribution was similar between both groups, patients with neurological sequelae or death at 6 months had a higher prevalence of coronary diseases and hypertension. CPC 1 patients had more often bystander CPR, shorter time to ROSC, lower lactate, NSE and CRP levels 48 h after ROSC.

Table 3 regroups the clinical and demographic data from the 511 patients of the multicenter TTM trial used for independent validation by qPCR. Among these, 234 patients fully recovered (CPC 1) and 277 had neurological sequelae or died (CPC 2–5). Patients in the CPC 2–5 group were older than patients in the CPC 1 group. There was no difference for BMI and sex. Patients in the CPC 2–5 group had more often hypertension. Patients in the CPC 1 group had more often bystander CPR, shorter time to ROSC, and lower lactate and NSE levels.

### Discovery phase

**Fig. 1:**
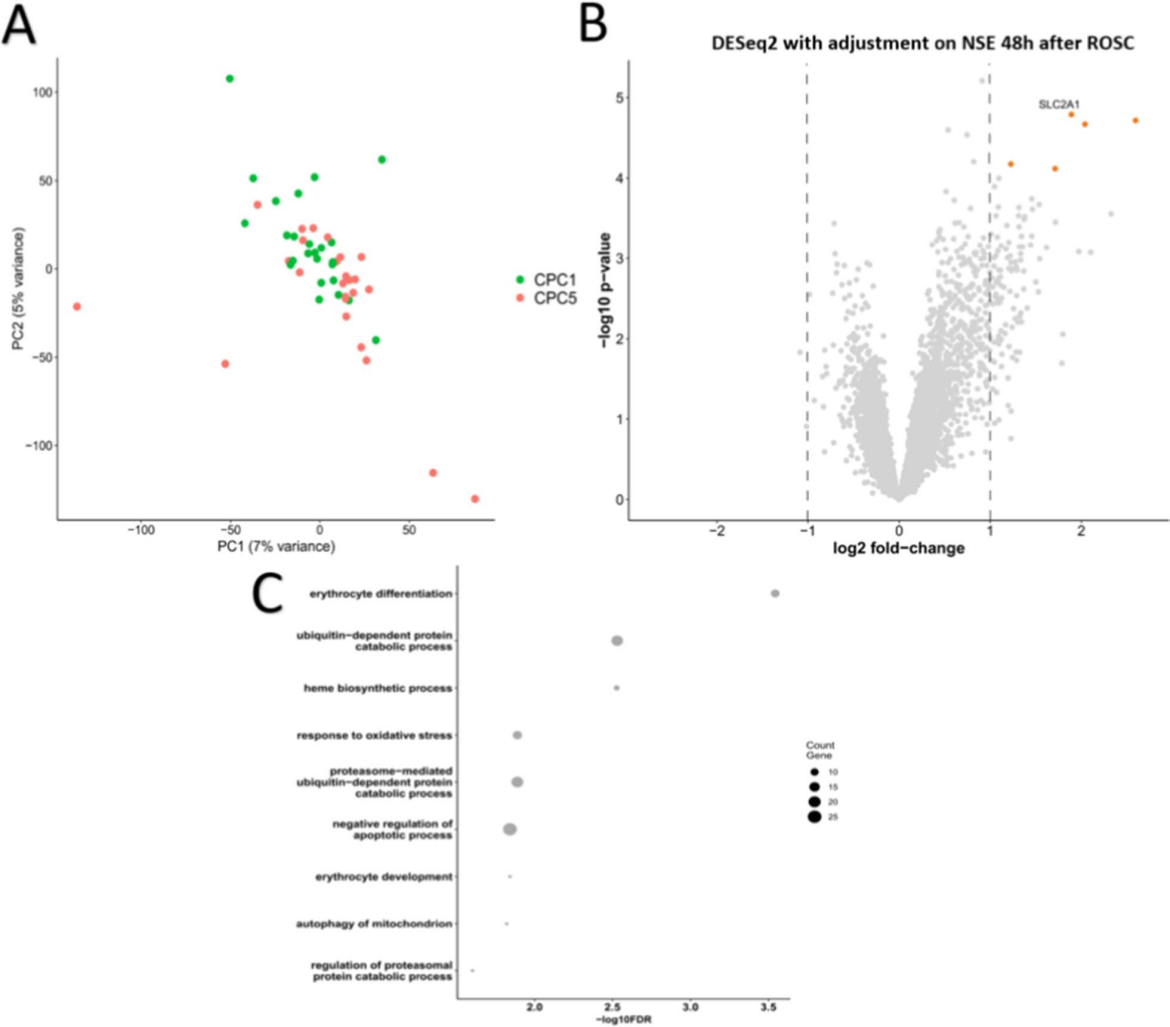
Transcriptomic differences between CPC 1 and CPC 5 patients after cardiac arrest. **A** Principal component analysis (PCA) of transcriptome profiles in CPC 1 and CPC 5. PCA was performed on variance-stabilized gene expression data (DESeq2) to assess global transcriptomic variation between survivors (CPC 1, green, *n* = 25) and non-survivors (CPC 5, red, *n* = 25). The first two principal components explained 7% and 5% of the variance, respectively. The analysis showed a modest separation between CPC 1 and CPC 5 groups, with CPC 5 samples displaying greater dispersion. **B** Volcano plot of differential gene expression between CPC 1 and CPC 5 patients. Differential expression analysis was performed using DESeq2 with neuron-specific enolase (NSE) levels at 48 h after ROSC, included as a covariate. With FDR < 0.05 and |log₂ fold change|≥ 1 as threshold, five genes including SLC2A1 were significantly upregulated in CPC 5 compared to CPC 1; SLC2A1 showed the strongest upregulation. No gene were significantly downregulated. **C** Functional pathways analysis underlying transcriptomic differences between CPC 1 and CPC 5. ORA was performed on 406 genes with *p* < 0.05 and |log_2_ fold change|> 0.5 between CPC 5 and CPC 1 patients. Ten biological processes were significantly enriched (FDR < 0.05), including response to oxidative stress, negative regulation of apoptotic process, erythrocyte differentiation, and heme biosynthetic process

We used direct RNAseq to analyze the transcriptomic profiles of the 50 North Pole patients included in the discovery phase (Table 1). An average of 4.5 ± 1.7 million of reads were obtained per sample. The alignment on human genome gave an average mapping rate of 98%. After filtering for genes with a minimum of 10 aligned reads in at least half samples of one group, we detected the expression of 7018 genes. Principal component analysis (PCA) on the detected genes illustrated only a modest separation between CPC 1 and CPC 5 patients. The two principal components explained a limited proportion of the variance (7% and 5%), indicating that global transcriptomic differences are subtle rather than strongly clustered. Samples from the CPC 5 group also showed greater dispersion (Fig. 1A). To identify genes differentially expressed between CPC 1 and CPC 5 groups, we performed differential gene expression using DESeq2 with NSE at 48 h as covariate, since NSE is a well-established biomarker of neuronal injury and may confound the association between gene expression and neurological outcome. Differential expression was assessed using a false discovery rate (FDR) < 0.05 and a |log₂ fold change|≥ 1 as threshold. Five genes, including SLC2A1, were upregulated in the CPC 5 group compared to the CPC 1 group (Fig. 1B). No gene was down-regulated with these criteria. Gene over-representation analysis (ORA) on 406 genes with *p* < 0.05 and log2 fold change > 0.5 or < − 0.5 identified 10 biological processes with FDR < 0.05, including response to oxidative stress and negative regulation of apoptotic process (Fig. 1C).

The most differentially expressed gene, SLC2A1, which encodes the glucose transporter GLUT1, emerged as the most compelling. SLC2A1 was upregulated in non-survivors with a log₂ fold change of 1.89 and an FDR of 0.028 after adjusting for 48 h-NSE. Given its critical role in glucose transport across the blood–brain barrier (which is disrupted after CA) and its regulation under hypoxic and ischemic conditions, SLC2A1 represents a biologically plausible and clinically relevant target which was selected for further investigation.

### Validation phase

**Fig. 2:**
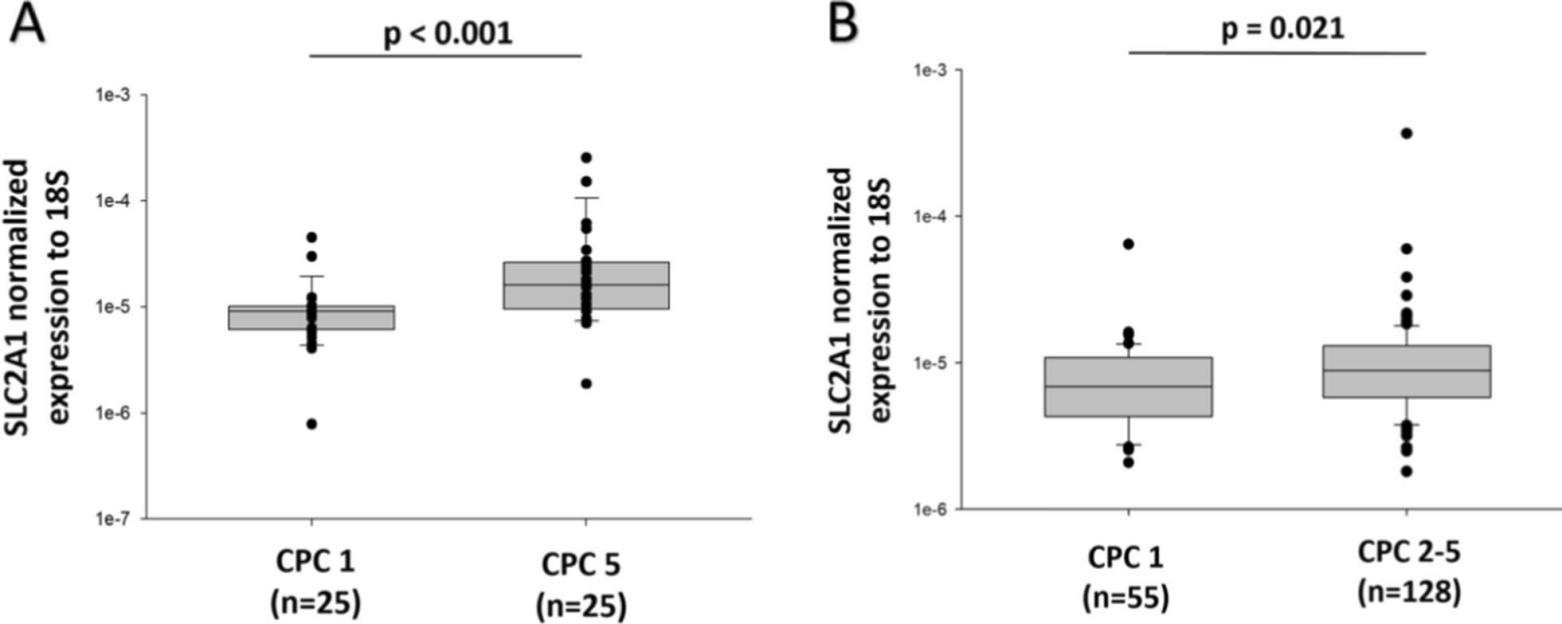
Expression of SLC2A1 measured by qPCR in the North Pole cohort. **A** Box plots showing the expression levels of SLC2A1 (solute carrier family 2 member 1) in the two groups of North Pole patients used in the discovery phase: 25 CPC 1 patients and 25 CPC 5 patients. SLC2A1 expression was significantly higher in CPC 5 patients. **B** Box plots showing the expression levels of SLC2A1 (solute carrier family 2 member 1) in 183 remaining patients of the North Pole cohort: 55 survivors without neurological sequelae at 6 months (CPC 1) and 128 patients with neurological sequelae or death (CPC 2–5). SLC2A1 expression remained significantly elevated in patients with neurological sequelae or death. Expression levels were measured by quantitative PCR (qPCR) and normalized to 18S ribosomal RNA. *P*-values are from Mann–Whitney *U* test

To validate the reliability of the RNAseq findings, the expression of SLC2A1 was first measured by qPCR in the discovery subset of 50 patients from the North Pole cohort, including 25 CPC 1 patients and 25 CPC 5 patients. As shown in Fig. 2A**,** SLC2A1 expression was higher in CPC 5 patients as compared to CPC 1 patients (*p* < 0.001), thus confirming RNAseq data.

SLC2A1 expression was then assessed by qPCR in the 183 remaining patients of the North Pole cohort. These 183 patients were categorized as CPC 1 (*n* = 55), CPC 2 (*n* = 23), CPC 3 (*n* = 17), CPC 4 (*n* = 5), and CPC 5 (*n* = 83). For the main analysis, patients were dichotomized into a group of survivors without neurological sequelae (CPC 1, *n* = 55) and a group with neurological sequelae or who died within 6 months (CPC 2–5, *n* = 128). As shown in Fig. 2B, SLC2A1 expression was higher in patients with neurological sequelae or death compared to survivors (*p* = 0.021). In addition to the global association with neurological sequelae or death (CPC 2–5), SLC2A1 expression was also significantly higher in patients who died (CPC 5, *n* = 83) compared with those who fully recovered (CPC 1). The corresponding analysis is shown in Supplementary Figure S1.

### Association between SLC2A1 expression and neurological outcome in the North Pole cohort

**Fig. 3:**
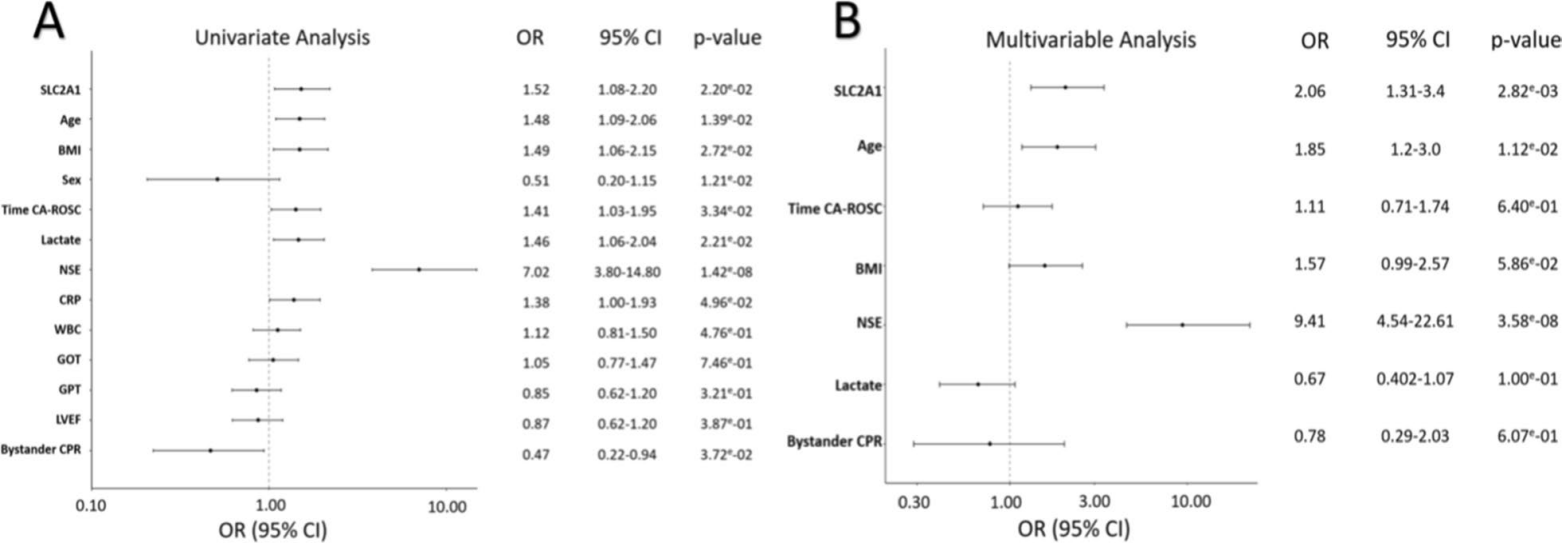
Univariate and multivariable logistic regression analysis for the prediction of neurological sequelae or death at 6 months after CA in 183 North Pole patients. **A** In univariate logistic regression, higher SLC2A1 expression was associated with an increased risk of neurological sequelae or death (CPC2–5). In multivariable analysis (**B**) adjusting for age, time CA-ROSC, BMI, NSE, lactate, and bystander CPR), SLC2A1 remained an independent predictor of neurological sequelae or death at 6 months after CA, along with older age and higher NSE levels. Forest plot showing odds ratios (OR), 95% confidence intervals (CI), and corresponding p-values for each variable are shown

Univariate and multivariable logistic regression analyses were performed to assess the association of SLC2A1 with neurological sequelae at 6 months post-CA. SLC2A1 was an univariate predictor of neurological sequelae or death (CPC 2–5), together with age, BMI, time to ROSC, NSE, lactate, CRP, and bystander CPR (Fig. 3A). Increased SLC2A1 expression was associated with a higher risk of neurological sequelae or death (odds ratio (OR) [95% confidence interval (CI)] 1.52 [1.08–2.20]. In multivariable analysis including significant predictors from the univariate model (age, time to ROSC, BMI, NSE, lactate, and bystander CPR), SLC2A1 remained independently associated with neurological sequelae or death (OR [95% CI] 2.06 [1.31–3.4], *p* = 2.82e-03), together with age (OR 1.85 [1.2–3.0], *p* = 1.12e-02) and NSE (OR 9.41 [4.54–22.61], *p* < 0.001) (Fig. 3B). These results indicate that SLC2A1 provides independent prognostic information beyond established clinical predictors of outcome after CA.

We then assessed the incremental predictive value of SLC2A1 in the 183 North Pole patients, dichotomized into survivors without neurological sequelae at 6 months (CPC 1) versus patients with neurological sequelae or death (CPC 2–5), using a baseline model including age, time to ROSC, BMI, NSE, lactate, and bystander CPR. The addition of SLC2A1 to the baseline model reduced the Akaike Information Criterion (AIC) from 164.87 to 156.18, indicating improved model fit. The likelihood ratio test showed a significant improvement in model performance (*p* = 0.001). The area under the receiver operating characteristic curve (AUC) increased from 0.864 to 0.884, although this difference did not reach statistical significance according to the DeLong’s test (*p* = 0.118). The reclassification analyses demonstrated a significant improvement of prediction with SLC2A1, with a net reclassification improvement (NRI) of 0.829 (*p* < 0.001) and an integrated discrimination improvement (IDI) of 0.05 (*p* = 0.003) when SLC2A1 was added to the baseline model. These results support the added prognostic value of SLC2A1 to conventional predictors in identifying patients at risk of neurological sequelae or death at 6 months after CA.

### Association between SLC2A1 expression and survival in the North Pole cohort

For 6-month survival analysis using Kaplan–Meier curves, the 183 patients of the North Pole cohort were dichotomized into high (95 patients) and low (88 patients) SLC2A1 expression groups based on an optimal cutoff defined by the Youden index (*Y* = 0.15). As shown in Figure S2, patients with high SLC2A1 expression had a tendency for a lower chance of survival compared to those with low expression (*p* = 0.057; log-rank test). To further assess the association between SLC2A1 and survival 6 months after CA, univariate and multivariable Cox proportional hazards analyses were performed. SLC2A1 was not significantly associated with survival in univariate analysis, while age, BMI, time CA-ROSC, lactate, NSE, and bystander CPR were significant predictors (Figure S3A). In multivariable Cox proportional hazards analysis (Figure S3B) including the clinical variables of the baseline model mentioned in the logistic regression above, age, BMI, NSE, and lactate were significant predictors of mortality. SLC2A1 expression was not independently associated with survival in this cohort.

### Independent validation in TTM trial cohort

**Fig. 4:**
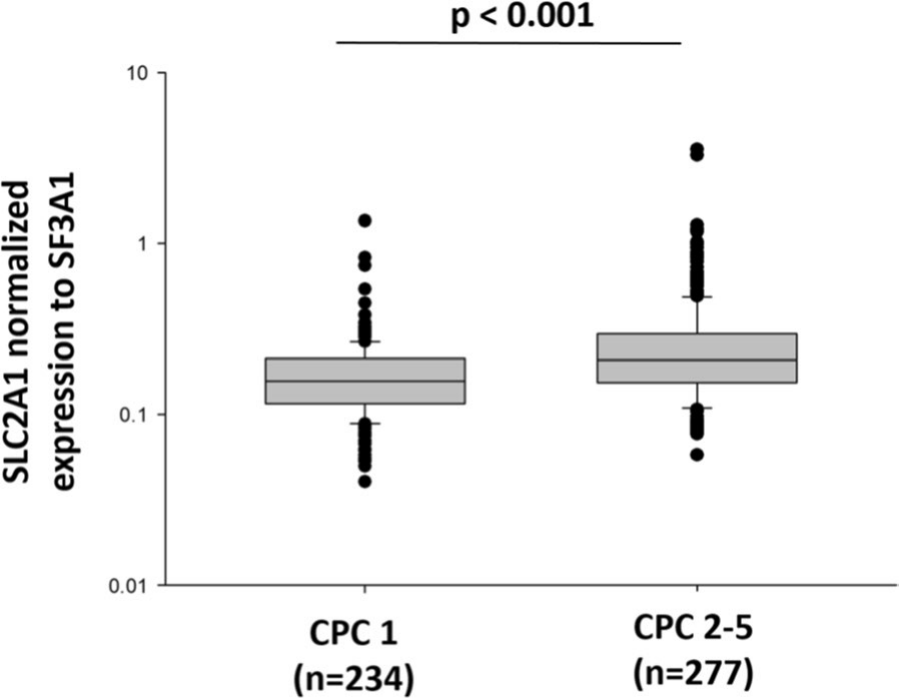
Expression of SLC2A1 by qPCR in 511 TTM trial patients. Box plots showing the relative expression levels of SLC2A1 (solute carrier family 2 member 1) in two groups of TTM trial patients. SLC2A1 expression measured by qPCR was significantly higher in patients with neurological sequelae or death (CPC 2–5, *n* = 277) compared to patients without neurological sequelae (CPC 1, *n* = 234). Data are normalized to 18S ribosomal RNA. *P*-value is from Mann–Whitney *U* test

We next examined the association of SLC2A1 with neurological outcome and survival after CA in an independent, multicenter cohort of CA patients (TTM trial). qPCR was performed on whole blood samples from 511 patients collected 48 after ROSC. 234 survivors completely recovered 6 months after CA (CPC 1) and 277 patients had neurological sequelae or death (CPC 2–5). Figure 4 shows that patients with neurological sequelae or death had a higher expression of SLC2A1 compared to survivors (*p* < 0.001), consistently with data obtained in the North Pole cohort.

**Fig. 5:**
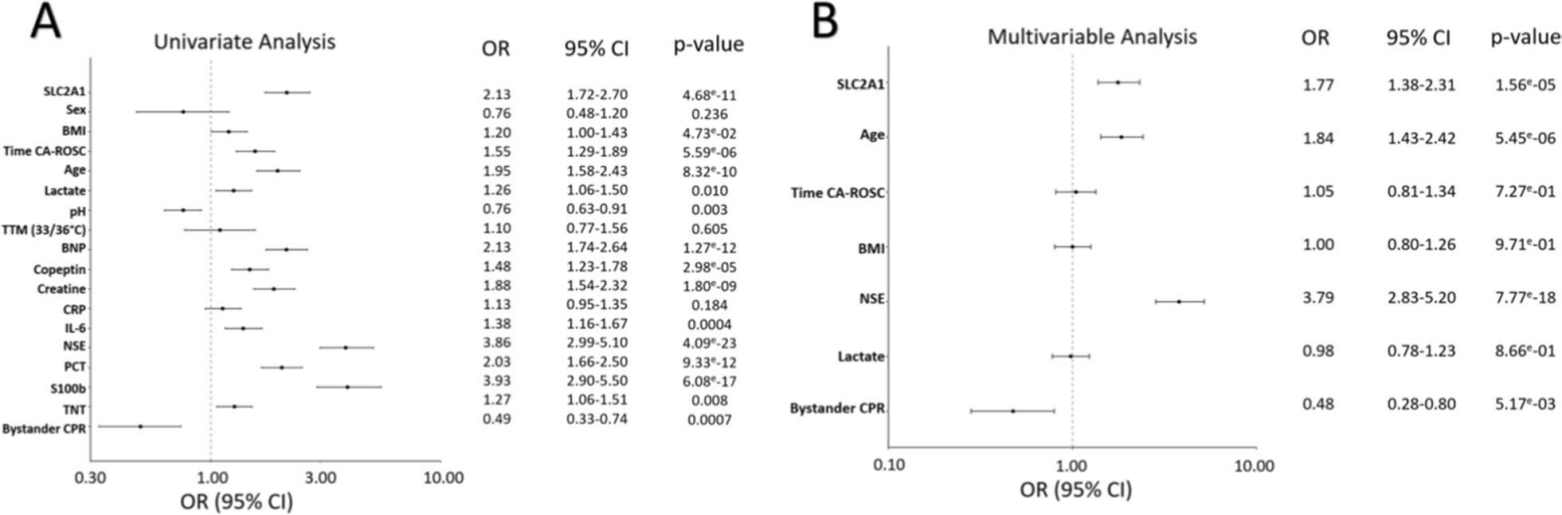
Univariate and multivariate logistic regression analysis for the prediction of neurological sequelae or death at 6 months after CA in 511 TTM trial patients. **A** In univariate analysis of 511 TTM patients, higher SLC2A1 expression was associated with increased risk of neurological sequelae or death (CPC 2–5), along with several established clinical and biochemical markers. **B** In multivariable analysis adjusting for age, time to ROSC, BMI, NSE, lactate, and bystander CPR, SLC2A1 remained an independent predictor, together with older age and higher NSE levels. SLC2A1 (solute carrier family 2 member 1), body mass index (BMI), time from cardiac arrest to return of spontaneous circulation (CA-ROSC), temperature targeted management (TTM), BNP (brain natriuretic peptide), copeptin (C-terminal segment of pre-provasopressin), IL-6 (interleukin-6), NSE (neuron-specific enolase), PCT (procalcitonin), S100b (S100 calcium-binding protein B), TNT (troponin T) and bystander cardiopulmonary resuscitation (CPR). Forest plots showing odds ratios (OR), 95% confidence intervals (CI), and corresponding *p*-values are shown for each variable

In univariate analyses, SLC2A1 was associated with neurological sequelae or death, as well as BMI, time CA-ROSC, age, lactate, pH, brain natriuretic peptide (BNP), copeptin, creatinine, interleukin-6 (IL-6), NSE, procalcitonin (PCT), S100 calcium-binding protein B (S100b), troponin T (TNT), and bystander CPR (Fig. 5A). The association of SLC2A1 with neurological outcome remained significant after adjustment with significant clinical variables in multivariable analyses (Fig. 5B), supporting the added value of SLC2A1 in the prognostic of neurological sequelae or death at 6 months after CA.

The incremental predictive value of SLC2A1 in 511 TTM trial patients, dichotomized into survivors (CPC 1) versus patients with neurological sequelae or death (CPC2–5), was assessed using a baseline model including age, time to ROSC, BMI, NSE, lactate, and bystander CPR. The addition of SLC2A1 to the baseline model reduced the AIC from 525.5 to 506, indicating improved model fit. The likelihood ratio test showed a significant improvement in model performance (*p* < 0.001). The AUC increased from 0.8398 to 0.8563 (*p* = 0.016; DeLong’s test). The reclassification analyses demonstrated a significant improvement, with an NRI of 0.364 (*p* < 0.001) and an IDI of 0.035 (*p* < 0.001) when SLC2A1 was added to the baseline model. These results validated the added prognostic value of SLC2A1 to conventional predictors in identifying patients at risk of neurological sequelae or death after CA.

In Kaplan–Meier survival analysis where the 511 patients of the TTM trial were dichotomized into high (286 patients) and low (225 patients) SLC2A1 expression groups based on the optimal cutoff determined by the Youden index (*Y* = 0.27), patients with high SLC2A1 expression had a lower probability of survival over the 6-month follow-up period compared to those with low expression (log-rank *p* < 0.0001) (Figure S4).

In univariate Cox proportional hazards analysis (Figure S5A), high levels of SLC2A1 were associated with increased mortality (HR [95% CI] 1.35 [1.21–1.50]), as well as increasing age, BMI, time CA-ROSC, lactate, BNP, copeptin, IL-6, NSE, PCT, S100b, and TNT. This association was lost after adjustment with age, BMI, NSE, lactate, and the absence of bystander CPR ((HR [95% CI] 1.01 [0.89–1.16]) (Figure S5B).

## Discussion

In this study, we identified SLC2A1 as a blood-based biomarker associated with neurological outcome after CA, with consistent upregulation observed in two independent cohorts and improvements in prognostic performance when integrated into clinical models.

The gene SLC2A1 encodes GLUT1, a glucose transporter highly expressed at the blood–brain barrier, in astrocytes and neurons [15]. GLUT1 is crucial for maintaining cerebral glucose uptake. Following CA, the brain experiences significant disruption of energy demand and glucose metabolism under hypoxic or ischemic conditions, leading to reduced glucose supply while energy demand remains high [16].

Our findings highlight the potential of transcriptomic profiling to uncover novel blood-based biomarkers and biological pathways involved in post-CA injury, and contribute to a growing evidence suggesting that circulating transcriptomic markers may provide complementary prognostic information to existing clinical variables.

Indeed, transcriptomic analysis of blood samples in CA is a relatively poorly addressed field, with only few studies conducted to date. One recent study reported a circular RNA, circular NFAT5, that could improve prediction of CA patient’s outcome [11] underscoring the growing recognition of RNA molecules as promising biomarkers and potential regulators of CA injury. In a sub-study of the TTM trial, elevated levels of the brain-enriched microRNA miR-124-3p were independently associated with poor neurological outcomes and reduced survival at 6 months [17]. Another study using genome-wide blood transcriptomic profiling in comatose survivors of out-of-hospital CA reported early differential expression patterns between patients with favorable versus unfavorable outcomes [18]. These studies support the potential of circulating transcriptomic markers to aid prognostication and provide biological insights into post-CA injury.

To investigate transcriptomic changes, we used Oxford Nanopore Technologies for direct RNA sequencing of peripheral blood samples collected 48 h after CA. This third-generation sequencing method allowed for the sequencing of native RNA molecules without amplification, providing long reads that improved transcript-level resolution [19].

Among the differentially expressed genes, SLC2A1 was significantly upregulated in patients dying following CA compared to those who fully recovered in the discovery cohort. We confirmed this finding with qPCR, which validated the increased SLC2A1 expression in dying patients but also in those, with neurological sequelae (CPC 2, 3 and 4). The association of comprehensive Nanopore sequencing and the sensitivity and specificity of qPCR strengthens the reliability of our findings and supports SLC2A1 as a potential marker of neurological injury after CA.

For the sake of reproducibility and to strengthen SLC2A1 potential as a biomarker, we used data from the independent multicenter TTM trial. This large and diverse population, recruited from 10 European centers, provided a broader clinical context and reduced the risk of center-specific bias. Consistent with our initial observations in the monocenter North Pole study, SLC2A1 remained significantly upregulated in patients suffering from neurological sequelae or death in the multicenter TTM trial.

We then investigated the incremental prognostic value of SLC2A1 using multivariable logistic regression and reclassification analyses. The inclusion of SLC2A1 expression in predictive models significantly improved the overall predictive performance in the two study cohorts. We observed a lower AIC, a higher AUC, and significant improvements in reclassification metrics, including NRI and IDI, when SLC2A1 was added to the baseline model. These results indicate that SLC2A1 provides independent prognostic information beyond conventional clinical variables. Indeed, existing biomarkers such as NSE and NfL provide valuable prognostic information after CA but have limitations. In our study, SLC2A1 added prognostic value beyond clinical variables including NSE and can be measured using standard molecular assays, suggesting its applicability in clinical settings.

However, when we assessed the association between SLC2A1 expression and survival over time, the results varied across cohorts. In the North Pole cohort, neither Kaplan–Meier survival analysis nor multivariable Cox proportional hazards models revealed a statistically significant association. In contrast, in the TTM trial, higher SLC2A1 expression was significantly associated with lower survival in Kaplan–Meier curves and in univariate Cox analysis. This association did not remain significant in the multivariable Cox model, suggesting the effect may be confounded by other clinical variables. These findings indicate that while SLC2A1 is strongly linked to neurological sequelae or death at 6 months, particularly in identifying patients with severe brain injury or death (CPC 2–5), its expression as a predictor of mortality or time to death within a 6-month period remains to be externally tested.

Thus, SLC2A1 appears to be more specific as a prognostic marker of neurological outcome rather than a general predictor of mortality. This distinction highlights the complexity of post-CA pathophysiology, where mechanisms driving neurological injury may differ from those influencing overall survival. While SLC2A1 appears more closely linked to brain-specific damage, its exact role in neurological injury remains unclear. Further functional studies are needed to better understand the biological role of SLC2A1 in hypoxic–ischemic brain injury.

The increased expression of SLC2A1 could reflect several processes such as an adaptive response where the brain attempts to restore glucose uptake and preserve neuronal survival under low oxygen conditions. Increased expression of SLC1A1 in whole blood samples may also reflect dysfunction of the blood–brain barrier or neuroinflammation. It may also indicate a compensation for impaired glucose transport where SLC2A1 may be upregulated to compensate for reduced glucose transport across the blood–brain barrier and other tissues, helping to maintain energy supply during hypoxia. Another possibility is that severe and prolonged hypoxia causes cell damage and death, particularly in cardiac muscle and endothelial cells, leading to the release of RNAs like SLC2A1 into the bloodstream. This leakage may be analogous to how cardiac biomarkers such as troponin enter the blood after heart injury [20]. Additionally, stressed cells may release membrane vesicles (e.g., exosomes) containing SLC2A1, further raising its circulating levels. Because SLC2A1 is widely expressed, not only in the brain but also in red blood cells and vascular endothelial cells [21], systemic hypoxia and reperfusion injury after CA likely cause widespread cellular stress and damage, possibly increasing SLC2A1 expression into the blood. Analysis were performed on whole-blood RNA, which includes a large proportion of red blood cells (RBCs). Because SLC2A1 is expressed in RBC membranes, these cells may contribute to the overall SLC2A1 expression detected in our samples. In addition, pathway enrichment analysis identified erythrocyte differentiation as one of the most significantly represented biological processes, suggesting that red blood cell-related transcripts may influence the observed gene expression patterns. Although the exact contribution of RBCs could not be quantified, this factor should be considered when interpreting whole-blood transcriptomic data. Together, these mechanisms align with key features of post-cardiac arrest pathophysiology such as the neuroenergetic failure, blood–brain barrier disruption, and systemic hypoxia, suggesting that elevated circulating SLC2A1 may represent a converging signal of impaired cerebral metabolism and widespread cellular stress.

This study has several limitations. First, the discovery cohort size was modest, and although validation in the large multicenter TTM trial strengthens our findings, differences in patient management across centers may have influenced results. Second, blood sampling was performed exclusively at 48 h after CA, where it may miss earlier expression dynamics. Assessing SLC2A1 kinetics at multiple time points (before and after 48 h post-ROSC) could reveal additional prognostic information. Third, while SLC2A1 was consistently associated with neurological sequelae or death, its relationship with survival was variable across cohorts and not independent in multivariable Cox analyses. Fourth, our analysis was based on peripheral blood samples, which while clinically, easily accessible, may not fully capture brain-specific injury pathways. Future studies using complementary biofluids such as cerebrospinal fluid could provide additional and deeper insights into the central nervous system injury mechanisms. Fifth, we did not perform protein-level or functional assays, and therefore cannot confirm that SLC2A1 mRNA levels reflect corresponding changes in GLUT1 protein abundance or activity. Sixth, the functional role of SLC2A1 in hypoxic–ischemic injury remains unclear, and its widespread expression beyond the brain may limit its specificity as a cerebral injury biomarker. Future studies integrating longitudinal sampling, complementary biofluids, and functional experiments are needed to clarify the biological relevance of SLC2A1 and its potential both as a biomarker and as a therapeutic target to improve neurological outcome after CA. Finally, our outcome classification (CPC 1 vs CPC 2–5) differs from the conventional distinction of CPC 1–2 as good outcome and CPC 3–5 as poor outcome. This approach may amplify group differences and limits direct comparison with studies that group CPC 2 patients with CPC 1, potentially obscuring mild neurological deficits within a group of fully recovered patients. We chose this categorization to distinguish preserved neurological function from any measurable impairment, without assigning labels such as ‘good’ or ‘poor’ outcome. In addition, although dysglycemia has been linked to patient’s outcome in the TTM trial [22], no time-matched glycemia data were available in the North Pole database, which prevents excluding confounding effects of systemic glucose level on SLC2A1 predictive value.

Overall, the consistent association of SLC2A1 with neurological sequelae or death across two independent cohorts underscores the robustness of this biomarker. This reproducibility strengthens the clinical relevance of SLC2A1 even though its mechanistic role in hypoxic–ischemic injury remains to be fully elucidated. Based on our data, SLC2A1 represents more a marker rather than a mediator of hypoxic–ischemic injury, as our study does not establish a causal role for GLUT1 in driving neurological damage. However, its consistent upregulation across two cohorts raises the possibility that altered glucose transport may not only reflect injury severity but could also participate in neuroenergetic failure. Distinguishing whether SLC2A1 is a marker or also a mediator will require targeted functional studies.

## Conclusion

Our study identifies SLC2A1 as a significant and independent prognostic biomarker to aid predicting neurological outcome after CA. We found consistent upregulation of SLC2A1 in patients with neurological sequelae or death across two independent cohorts, North Pole and TTM trial. Adding SLC2A1 expression to clinical prediction models significantly improved their prognostic performance for neurological outcome. The exact biological role of elevated SLC2A1 in hypoxic–ischemic brain injury requires further investigation.

## Supplementary Information

Supplementary Material 1. Figure S1. Expression of SLC2A1 by qPCR in 55 CPC 1 and CPC 5 North Pole patients. Box plots showing the expression levels of SLC2A1 (solute carrier family 2 member 1) in North Pole patients, including 55 CPC 1 patients and 83 CPC 5 patients. Expression levels were measured by quantitative PCR (qPCR) and normalized to 18S ribosomal RNA. *P*-value is from Mann–Whitney *U* test. Figure S2. Kaplan–Meier survival analysis stratified by SLC2A1 expression levels using the Youden index-derived cutoff in 183 North Pole patients. Patients were divided into high (95 patients) and low (88 patients) SLC2A1 expression groups based on the optimal cutoff determined by the Youden index (*Y* = 0.15). The log-rank p-value is indicated. Shaded areas represent 95% confidence intervals. Figure S3. Univariate and multivariable Cox proportional hazards analysis for the prediction of death at 6 months after CA in 183 North Pole patients. **A** Univariate, **B** Multivariable Cox proportional hazard analysis. Forest plot displays hazard ratios (HR) and 95% confidence intervals (CI) for each variable analyzed individually. Body mass index (BMI), time from cardiac arrest to return of spontaneous circulation (Time CA-ROSC), neuron-specific enolase (NSE), and bystander cardiopulmonary resuscitation (CPR), SLC2A1, (solute carrier family 2 member 1) C-reactive protein (CRP), white blood cell count (WBC), aspartate transaminase (GOT), alanine transaminase (GPT), left ventricular ejection fraction (LVEF). Figure S4. Kaplan–Meier survival analysis stratified by SLC2A1 expression levels using the Youden index-derived cutoff in 511 TTM trial patients. Patients were divided into high (286 patients) and low (225 patients) SLC2A1 expression groups based on the optimal cutoff determined by the Youden index (*Y* = 0.27). The log-rank p-value is indicated. Shaded areas represent 95% confidence intervals. Figure S5. Univariate and multivariable Cox proportional hazards model for the prediction of death at 6 months after CA in 511 TTM trial patients. **A** Univariate, **B** multivariable logistic regression. Forest plots show the hazard ratios (HR), 95% confidence intervals (CI), and *p*-values for each variable analyzed independently. Body mass index (BMI), time from cardiac arrest to return of spontaneous circulation (CA-ROSC), targeted temperature management (TTM), BNP (brain natriuretic peptide), copeptin (C-terminal segment of pre-provasopressin), IL-6 (interleukin-6), NSE (neuron-specific enolase), PCT (procalcitonin), S100b (S100 calcium-binding protein B), TNT (troponin T) and bystander cardiopulmonary resuscitation (CPR)

## Data Availability

The data contain sensitive patient information and are therefore not publicly available.

## Abbreviations

AIC: Akaike Information Criterion
AUC: Area under the receiver operating characteristic curve
BBB: Blood–brain barrier
BMI: Body mass index
BNP: Brain natriuretic peptide
CA: Cardiac arrest
CI: Confidence interval
CPC: Cerebral Performance Category
CPR: Cardiopulmonary resuscitation
CRP: C-reactive protein
CT: Computed tomography
DAVID: Database for Annotation, Visualization, and Integrated Discovery
EEG: Electroencephalography
FDR: False discovery rate
GLUT1: Glucose transporter 1
HR: Hazard ratio
ICU: Intensive care unit
IDI: Integrated discrimination improvement
IL-6: Interleukin-6
LRT: Likelihood ratio test
MRI: Magnetic resonance imaging
mRS: Modified Rankin scale
NSE: Neuron-specific enolase
NRI: Net reclassification improvement
ONT: Oxford Nanopore Technologies
OR: Odds ratio
PCA: Principal component analysis
PCT: Procalcitonin
qPCR: Quantitative polymerase chain reaction
RNAseq: RNA sequencing
ROSC: Return of spontaneous circulation
S100B: S100 calcium-binding protein B
SLC2A1: Solute carrier family 2 member 1
SSEP: Somatosensory evoked potentials
TTM: Targeted temperature management
UCH-L1: Ubiquitin carboxy-terminal hydrolase L1
