# Supplementary material for: Direct RNA sequencing identified solute carrier family 2 member 1 to improve neurological outcome prediction after cardiac arrest
Source: Intensive Care Med Exp. 2026 Jan 7;14:3. doi: 10.1186/s40635-025-00851-8 (PMC12779862; doi:10.1186/s40635-025-00851-8)
Supplement: Supplementary file 1 — Supplementary Material 1. Figure S1. Expression of SLC2A1 by qPCR in 55 CPC 1 and CPC 5 North Pole patients. Box plots showing the expression levels of SLC2A1 (solute carrier family 2 member 1) in North Pole patients, including 55 CPC 1 patients and 83 CPC 5 patients. Expression levels were measured by quantitative PCR (qPCR) and normalized to 18S ribosomal RNA. P-value is from Mann–Whitney U test. Figure S2. Kaplan–Meier survival analysis stratified by SLC2A1 expression levels using the Youden index-derived cutoff in 183 North Pole patients. Patients were divided into high (95 patients) and low (88 patients) SLC2A1 expression groups based on the optimal cutoff determined by the Youden index (Y = 0.15). The log-rank p-value is indicated. Shaded areas represent 95% confidence intervals. Figure S3. Univariate and multivariable Cox proportional hazards analysis for the prediction of death at 6 months after CA in 183 North Pole patients. A Univariate, B Multivariable Cox proportional hazard analysis. Forest plot displays hazard ratios (HR) and 95% confidence intervals (CI) for each variable analyzed individually. Body mass index (BMI), time from cardiac arrest to return of spontaneous circulation (Time CA-ROSC), neuron-specific enolase (NSE), and bystander cardiopulmonary resuscitation (CPR), SLC2A1, (solute carrier family 2 member 1) C-reactive protein (CRP), white blood cell count (WBC), aspartate transaminase (GOT), alanine transaminase (GPT), left ventricular ejection fraction (LVEF). Figure S4. Kaplan–Meier survival analysis stratified by SLC2A1 expression levels using the Youden index-derived cutoff in 511 TTM trial patients. Patients were divided into high (286 patients) and low (225 patients) SLC2A1 expression groups based on the optimal cutoff determined by the Youden index (Y = 0.27). The log-rank p-value is indicated. Shaded areas represent 95% confidence intervals. Figure S5. Univariate and multivariable Cox proportional hazards model for the prediction of d [file 40635_2025_851_MOESM1_ESM.docx]

**Supplementary figures:**


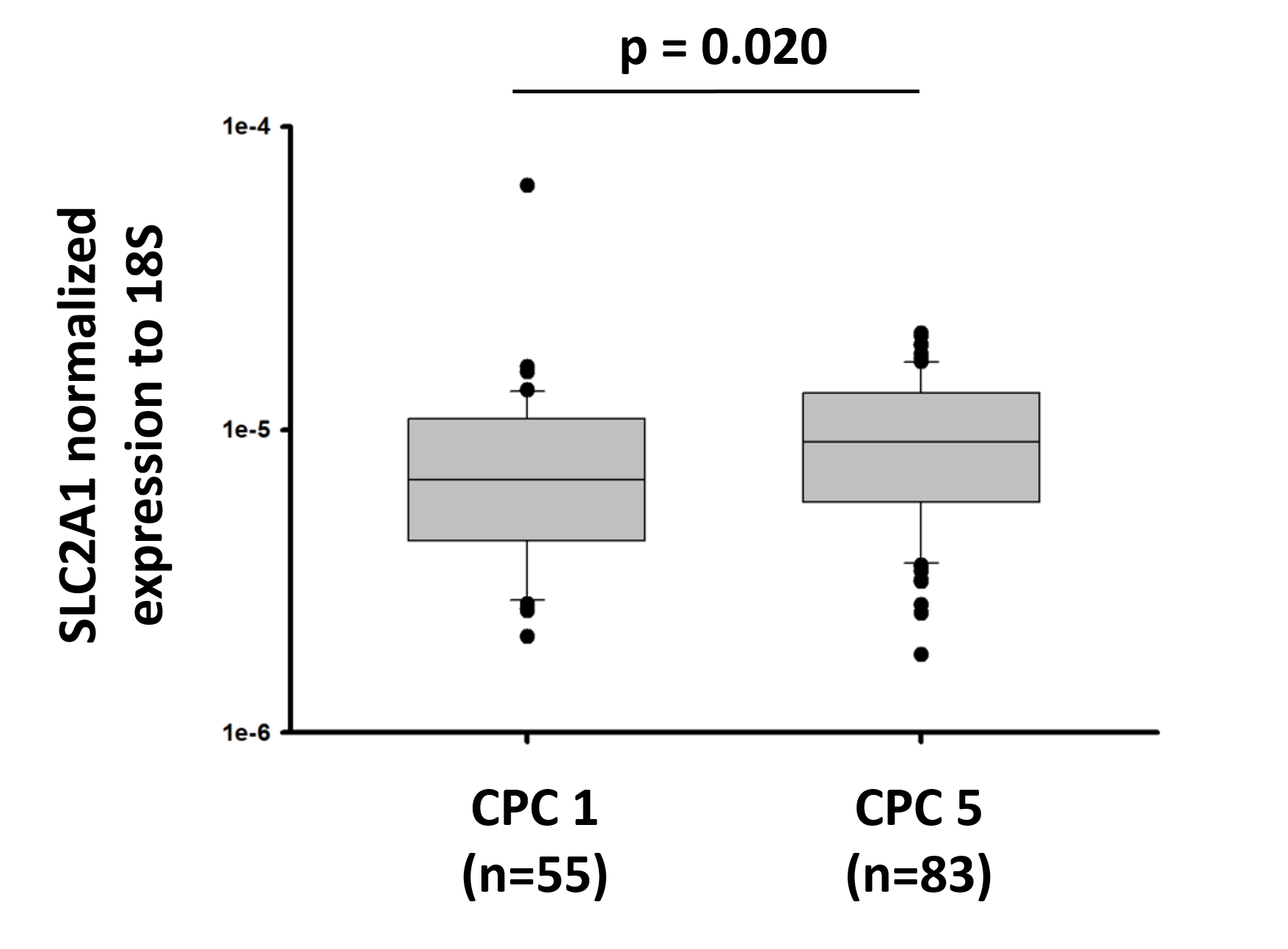


**Figure S1. Expression of SLC2A1 by qPCR in 55 CPC 1 and CPC 5 North Pole patients.** Box plots showing the expression levels of SLC2A1 (solute carrier family 2 member 1) in North Pole patients, including 55 CPC 1 patients and 83 CPC 5 patients. Expression levels were measured by quantitative PCR (qPCR) and normalized to 18S ribosomal RNA. P-value is from Mann–Whitney U test.


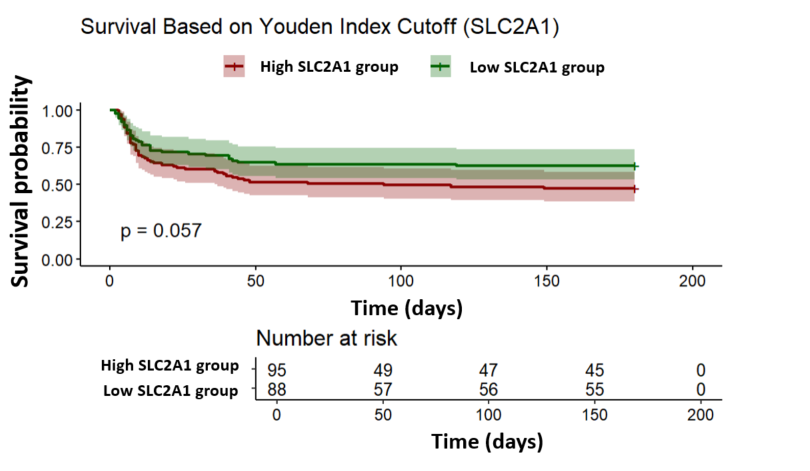


**Figure S2.** **Kaplan–Meier survival analysis stratified by SLC2A1 expression levels using the Youden index-derived cutoff in 183 North Pole patients.** Patients were divided into high (95 patients) and low (88 patients) SLC2A1 expression groups based on the optimal cutoff determined by the Youden index (Y= 0.15). The log-rank p-value is indicated. Shaded areas represent 95% confidence intervals.


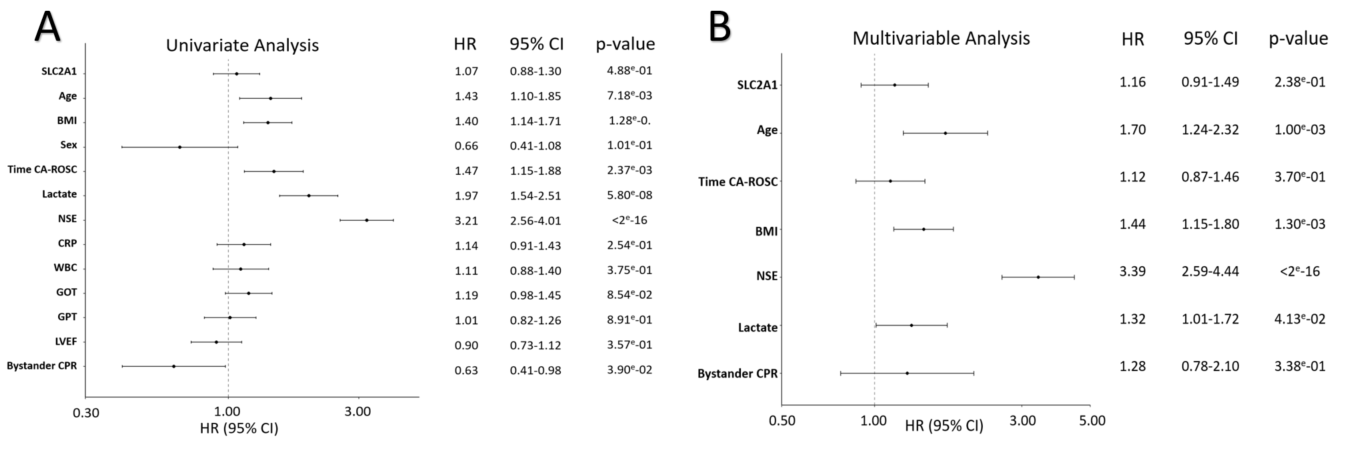


**Figure S3.** **Univariate and multivariable Cox proportional hazards analysis for the prediction of death at 6 months after CA in 183 North Pole patients. (A) Univariate, (B) Multivariable Cox proportional hazard analysis.** Forest plot displays hazard ratios (HR) and 95% confidence intervals (CI) for each variable analyzed individually. Body mass index (BMI), time from cardiac arrest to return of spontaneous circulation (Time CA-ROSC), neuron-specific enolase (NSE), and bystander cardiopulmonary resuscitation (CPR), SLC2A1, (solute carrier family 2 member 1) C-reactive protein (CRP), white blood cell count (WBC), aspartate transaminase (GOT), alanine transaminase (GPT), left ventricular ejection fraction (LVEF).


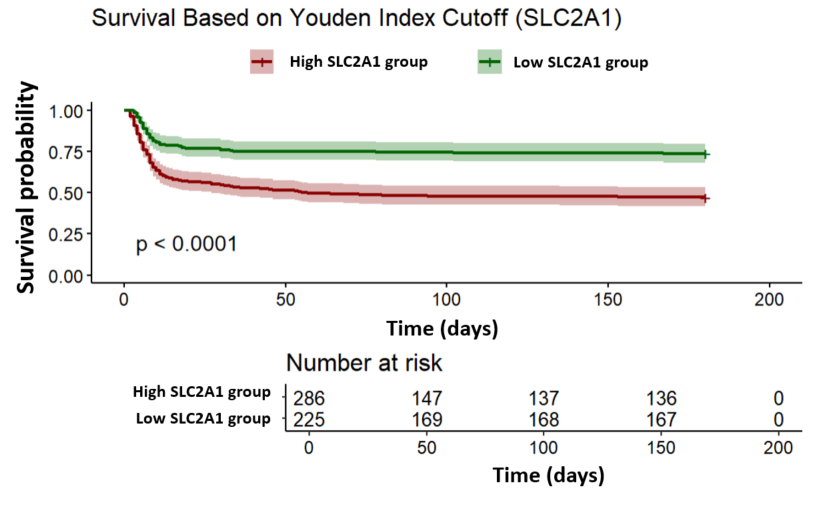


**Figure S4.** **Kaplan–Meier survival analysis stratified by SLC2A1 expression levels using the Youden index-derived cutoff in 511 TTM trial patients.** Patients were divided into high (286 patients) and low (225 patients) SLC2A1 expression groups based on the optimal cutoff determined by the Youden index (Y= 0.27). The log-rank p-value is indicated. Shaded areas represent 95% confidence intervals.


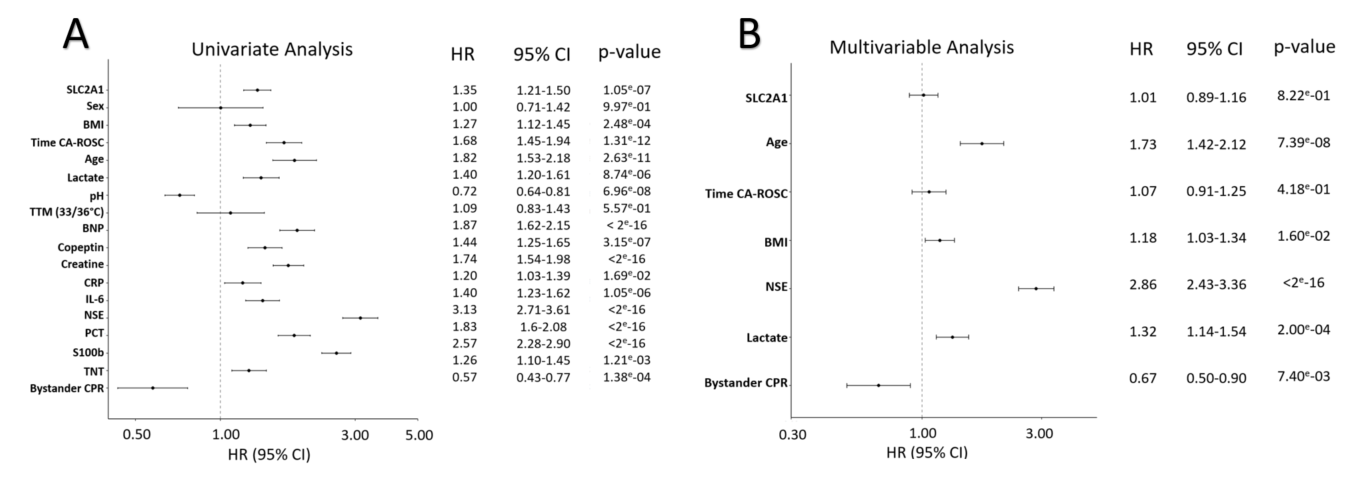


**Figure S5. Univariate and multivariable Cox proportional hazards model for the prediction of death at 6 months after CA in 511 TTM trial patients. (A) Univariate, (B) multivariable logistic regression.** Forest plots show the hazard ratios (HR), 95% confidence intervals (CI), and p-values for each variable analyzed independently. Body mass index (BMI), time from cardiac arrest to return of spontaneous circulation (CA-ROSC), targeted temperature management (TTM), BNP (brain natriuretic peptide), copeptin (C-terminal segment of pre-provasopressin), IL-6 (interleukin-6), NSE (neuron-specific enolase), PCT (procalcitonin), S100b (S100 calcium-binding protein B), TNT (troponin T) and bystander cardiopulmonary resuscitation (CPR).
